# First evidence of Limoniidae (Diptera: Nematocera) in French amber from Oise

**DOI:** 10.1038/s41598-025-99045-1

**Published:** 2025-04-25

**Authors:** Katarzyna Kopeć, Wiesław Krzemiński, Iwona Kania-Kłosok, Maksymilian Syratt, André Nel, Agnieszka Soszyńska

**Affiliations:** 1 Institute of Systematics and Evolution of Animals, Polish Academy of Sciences, Sławkowska 17, 31-016 Kraków, Poland; 2 Institute of Biology, University of Rzeszów, Rzeszów, Poland Zelwerowicza 4, 35‑601; 3 Department of Invertebrate Evolution, Institute of Zoology and Biomedical Research, Faculty of Biology, Jagiellonian University, Gronostajowa 9, 30-387 Kraków, Poland; 4 Institut de Systématique, Évolution, Biodiversité (ISYEB) Muséum national d’Histoire naturelle, CNRS, Sorbonne Université, EPHE, Université des Antilles, CP50, 57 rue Cuvier, 75005 Paris, France; 5 Department of Invertebrate Zoology and Hydrobiology, Faculty of Biology and Environmental Protection, University of Lodz, Banacha 12/16, 90-237 Łódź, Poland

**Keywords:** Insects, Fossil flies, Northern France, New species, Evolution, Paleobiodiversity, Palaeontology, Taxonomy, Palaeoclimate

## Abstract

This paper describes three new species found as inclusions in Early Eocene amber from Oise (northern France). Two of these species belong to the genus *Cheilotrichia*: *Cheilotrichia oisensis* Kopeć, Krzemiński & Kania-Kłosok sp. nov, *Cheilotrichia gallica* Kopeć, Krzemiński & Kania-Kłosok sp. nov., while one represents the genus *Dicranomyia: Dicranomyia* (*Dicranomyia*) *podenasi* Kopeć, Krzemiński & Kania-Kłosok sp. nov. This marks the first discovery of Limoniidae representatives in Oise amber, the oldest known Eocene resin, dating back approximately 55–53 Ma (Early Eocene, Ypresian). Our analysis indicates that the species composition of Limoniidae (Diptera, Nematocera) in Oise amber differs from that in Baltic amber. Despite their similar geographical origins, these ambers were produced in different periods and by different trees. The study of Oise amber provides valuable insights into the fauna of the earliest Eocene. These new discoveries contribute to our understanding of the evolutionary history of Limoniidae, particularly during the Early Eocene.

## Introduction

**Fig. 1:**
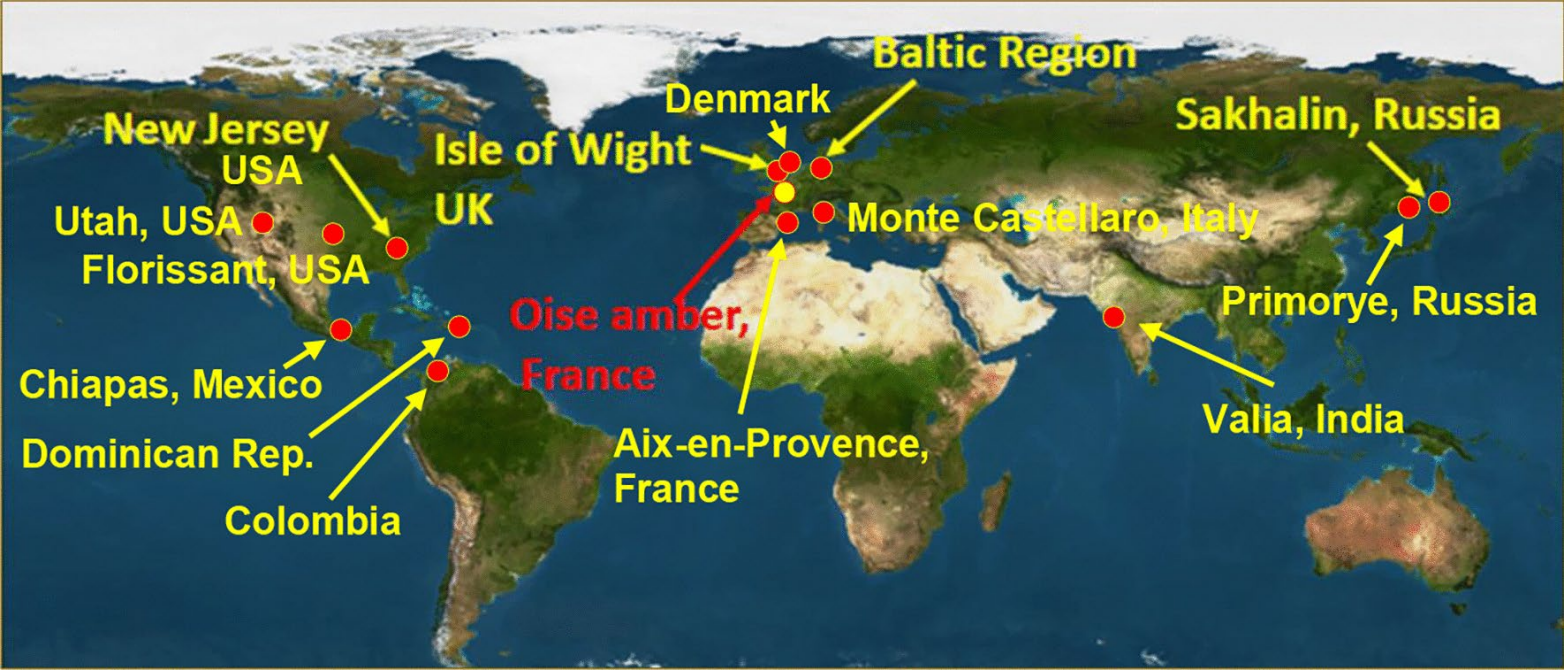
Map of Eocene outcrops from where species of *Cheilotrichia* and *Dicranomyia* were described (https://paleobiodb.org/navigator/). The yellow dot marks the new locality, while red dots indicate previously published localities.

The family Limoniidae (Diptera, Nematocera) is among the most diverse groups of flies in the modern fauna. With over 10,000 described species, it represents the largest family within the suborder Tipulomorpha and one of the largest among all Nematocera^1^. Most limoniids are small flies characterised by slender bodies, long legs, and elongated antennae. Adults are typically found in shaded, moist environments near water, where they play a crucial role as pollinators and as an essential food source for predators. Limoniidae is currently divided into seven subfamilies, including three extinct: Architipulinae Handlirsch, 1906, Eotipulinae Handlirsch, 1906, and Drinosinae Krzemiński, Kania-Kłosok, Krzemińska, Ševčík, Soszyńska-Maj, 2021, and four extant: Limnophilinae Bigot, 1854, Chioneinae Rondani, 1841, Dactylolabinae Alexander 1920, and Limoniinae Speiser, 1909^2^. Fossil evidence suggests that members of this family have existed since the Late Triassic (ca. 210 Ma), with the Architipulinae subfamily representing its earliest known lineage. A significant radiation of limoniids occurred during the Early Jurassic, as evidenced by numerous fossil records from this period^2–11^. While many genera appeared during the Jurassic and Lower Cretaceous, most did not survive into the present, such as *Architipula* Handlirsch, 1906 and *Grimenia* Krzemiński & Zessin, 1990^4,12^ or survived as relict group—*Chilelimnophila* Alexander, 1968^13^. Another milestone in the evolutionary history of Limoniidae occurred during the Eocene, a time characterised by the formation of extensive amber deposits. Eocene resins have been discovered in various locations, including Oise, Sakhalin, the Baltic region, Ukraine, Bitterfeld, India, and China. Spanning from 56 to 34 million years ago, the Eocene epoch began with the Paleocene-Eocene Thermal Maximum (PETM), a period of exceptionally high temperatures. Although temperatures gradually decreased over time, the warm climate of the Eocene significant influenced the evolution of fauna across Europe and North America. The largest Eocene amber deposits are found in Europe (Fig. 1), and the exceptionally well-preserved specimens they contain allow for detailed comparisons between extinct and extant species and provide valuable insight into the evolutionary history of these insects. This high-quality fossils enable precise reconstruction of body morphology, including critical diagnostic structures such as the hypopygium, which is essential for species identification.

This study provides descriptions of three new species of limoniid flies discovered for the first time in French amber from Oise (Fig. 1). Two of these species belong to the genus *Cheilotrichia*: *Cheilotrichia oisensis* Kopeć, Krzemiński & Kania-Kłosok sp. nov, *Cheilotrichia gallica* Kopeć, Krzemiński & Kania-Kłosok sp. nov., while the third species represents the genus *Dicranomyia*—*Dicranomyia* (*Dicranomyia*) *podenasi* Kopeć, Krzemiński & Kania-Kłosok sp. nov. The findings highlight differences in the species composition of Limoniidae (Diptera, Nematocera) between Oise amber and Baltic amber, contributing to a better understanding of their biogeography and evolutionary dynamics.

### Systematic paleontology

Order: Diptera Linnaeus, 1758.

Family: Limoniidae Speiser, 1909.

Subfamily: Chioneinae Rondani, 1841.

Genus: *Cheilotrichia* Rossi, 1848^14^.

Type species *Erioptera imbuta* Meigen, 1818^15^, by monotypy.

Subgenus: *Empeda* Osten Sacken, 1869^16^.

Type species *Empeda stigmatica* Osten Sacken, 1869; by subsequent designation of Coquillett 1910: 533^17^.

*Cheilotrichia* (*Empeda*) *oisensis* Kopeć, Krzemiński & Kania-Kłosok sp. nov.

(Fig. 2A–F).

**Fig. 2:**
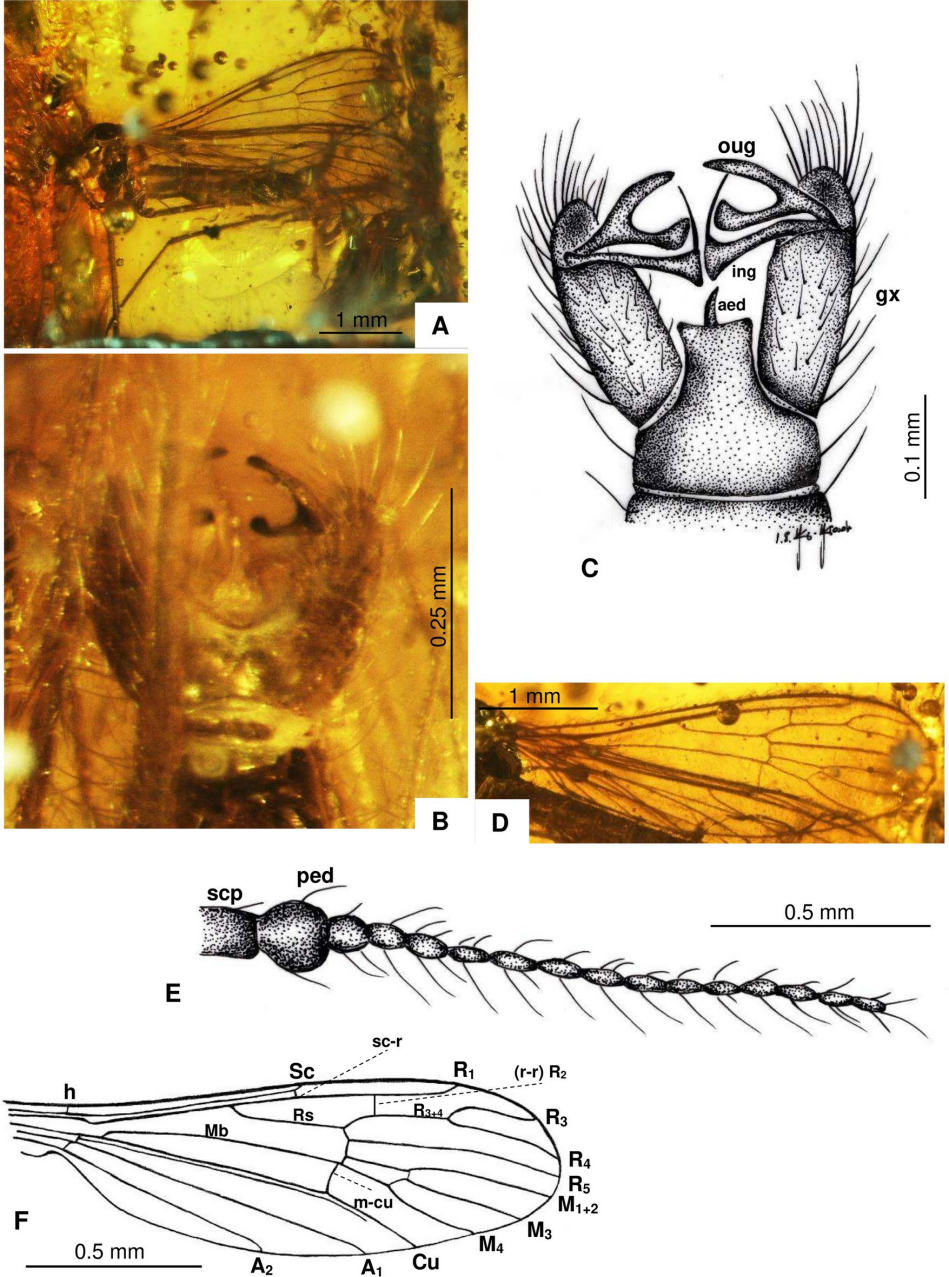
*Cheilotrichia* (*Empeda*) *oisensis* Kopeć, Krzemiński & Kania-Kłosok sp. nov., (**A**–**F**) holotype MNHN.F.A.97848 (male). (**A**) Habitus, lateral view; (**B**) Hypopygium, ventral view; (**C**) Hypopygium, dorsal view, drawing; (**D**) Wing; (**E**) Antenna, drawing; (**F**) Wing, drawing.

LSID urn:lsid:zoobank.org:act:8CEFB52D-4274-42CB-AA22-9F81CA0DF07F

#### Diagnosis

Vein Rs longer than R_3+4_; outer gonostylus widely forked, upper arm of fork slender with rather sharp tip, lower arm of fork significantly widened at tip and slightly convex in middle part; inner gonostylus strongly widened at the end with long, thick bristles at its tip.

#### Etymology

Species epithet after Oise River in northern France.

#### Material examined

Holotype:

MNHN.F.A.97848 (male), additional material:

MNHN.F.A.97849 (male), MNHN.F.A.97850 (male) (both specimens in the same piece of amber), Muséum national d’Histoire naturelle, Paris, France.

#### Age and occurrence

Eocene, Oise (French) amber, (ca. 55–53 Ma) (Early Eocene, Ypresian, Sparnacian)^18–21^, during the Eocene Thermal Maximum ETM-2 and subsequent hyperthermals^22,23^.

#### Description

Body small (Fig. 2A), approximately 2.90 mm long (holotype), 2.07 mm long (addit. mat.), wing length 3.16 mm (holotype), 3.05 mm (addit. mat.), width 0.93 mm (holotype), 0.75–0.98 mm (addit. mat.). Head and thorax dark, almost black.

*Head* (Fig. 2A): antenna short, 1.2 mm long (holotype), 0.78 mm long (addit. mat.), 16 antennomeres (Fig. 2E), antenna not reaching base of wing when bent toward back of body; scape elongate and cylindrical; pedicel wide and barrel-like; remaining antennomeres slightly elongate and oval; each flagellomere with four elongate setae, significantly longer than length of antennomere on which they arise; palpus short, with last segment equal in length to the penultimate one.

*Wings* (Fig. 2D,F): covered with elongate setae; vein Sc reaching approximately 0.5 × length of wing; sc-r near the apex of Sc, one length ahead of Sc tip; R_1_ relatively short, extending to the wing margin just behind R_3+4_ branch; Rs slightly longer than R_3+4_ and exceeds R_3_ by 0.25 × its length; vein R_3+4_ shorter than vein R_3_; crossvein r–r (R_2_) just beyond half length of R_1_; d-cell small and slightly widened at the apex; crossvein m-cu nearly straight, just before Mb branches into M_1+2_ and M_3+4_; A_1_ and A_2_ elongate, almost straight, and slightly curved at their tip.

*Legs* (Fig. 2A): covered with elongate, not numerous setae.

*Hypopygium* (Fig. 2B,C) 0.37 mm long (holotype), 0.32–0.39 mm long (addit. mat.), gonocoxite elongated, 2.5 × longer than wide, covered with setae, with bundle of elongate setae on its dorsal side; outer gonostylus widely forked, with upper arm of the bifurcation slender and sharply pointed, lower arm considerably expanded at the tip and slightly convex in its middle part; inner gonostylus strongly widened at the end, terminates in long, thick bristles; aedeagus straight, slender, and sharply tipped.

*Cheilotrichia* (*Empeda*) *gallica* Kopeć, Krzemiński & Kania-Kłosok sp. nov.

(Fig. 3A–F).

**Fig. 3:**
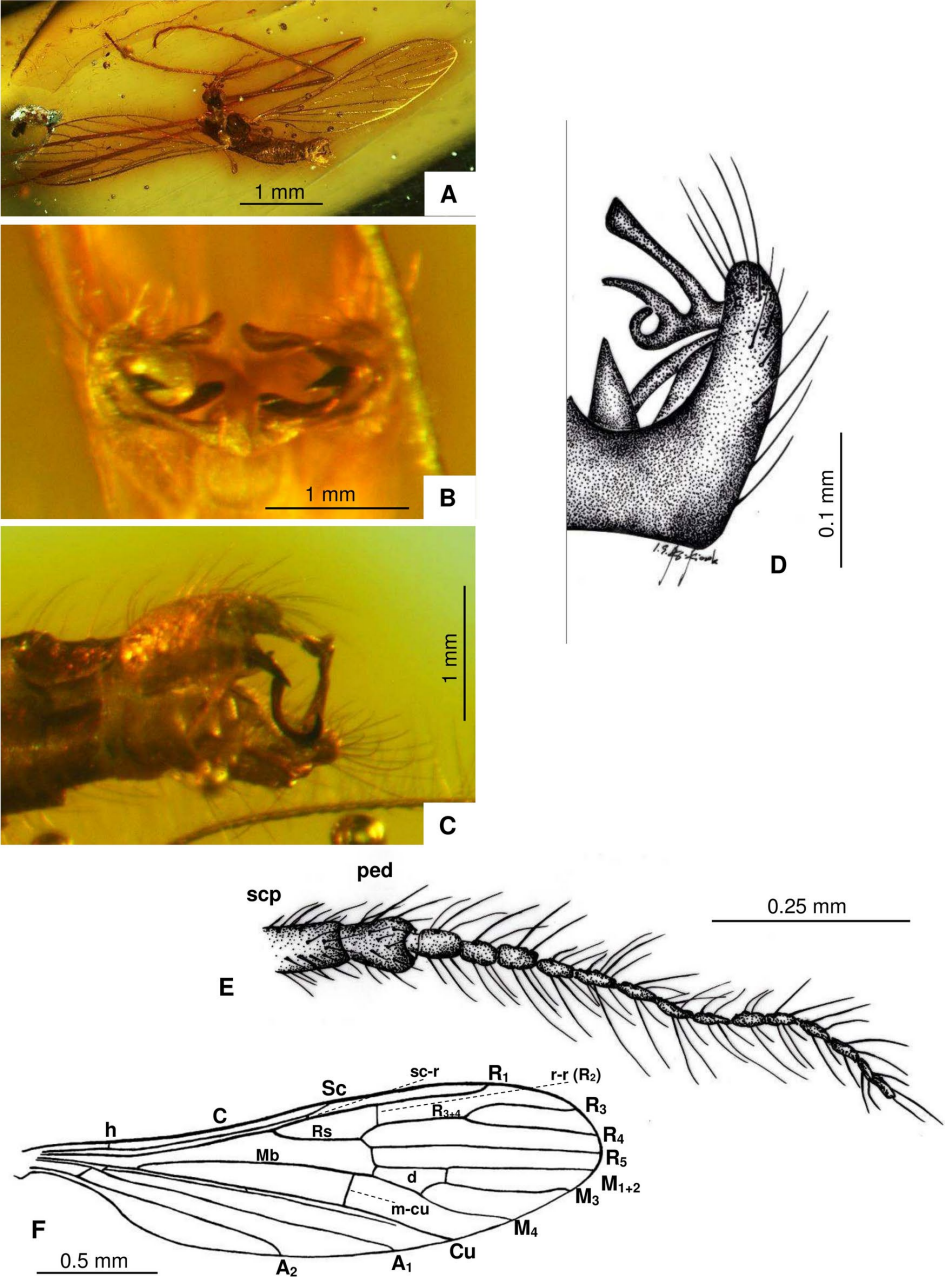
*Cheilotrichia* (*Empeda*) *gallica* Kopeć, Krzemiński & Kania-Kłosok sp. nov., (**A**–**F**) holotype MNHN.F.A.97852 : (**A**) Habitus, latero-ventral view; (**B**) Hypopygium, frontal view; (**C**) Hypopygium, latero-dorsal view; (**D**) Hypopygium, ventral view (part), drawing; (**E**) Antenna, drawing; (**F**) Wing, drawing.

#### Diagnosis

Vein Rs shorter than both R_3+4_ and R_3_; outer gonostylus furcates into three long processes, the last of which is strongly curled towards the second process; inner gonostylus strongly widened at the end, terminates in long, thick bristles.

#### Etymology

The species is named after the Latin name for France, the country of origin of the specimen.

#### Material examined

Holotype, MNHN.F.A.97852 (male), additional material, MNHN.F.A.97851 (male) (both specimens preserved in one piece of amber), housed in the Muséum national d’Histoire naturelle, Paris, France.

#### Age and occurrence

Eocene, Oise (French) amber, (ca. 55–53 Ma) (Early Eocene, Ypresian Sparnacian)^18–21^, during the Eocene Thermal Maximum ETM-2 and subsequent hyperthermals^22,23^.

#### Description

Body small (Fig. 3A), approximately 2 mm long, wing length 2.4 mm. Head and thorax dark, almost black.

*Head* (Fig. 3A): antenna short, 16 antennomeres (Fig. 3E), antenna reaching base of wing when bent toward back of body, scape elongate and cylindrical; pedicel wide, elongate, and barrel-like; first and second flagellomeres slightly widened at apex, remaining flagellomeres oval, slightly elongate; each flagellomere bears a few elongate setae, much longer than length of antennomeres on which they arise; palpus short, with last segment equal in length to penultimate one.

*Wings* (Fig. 3A,F): covered with elongate setae; tip of vein Sc beyond 0.5 × wing length, at approximately 0.6 × its length; sc-r far from tip of Sc, positioned at four times its length before end of Sc; R_1_ relatively long, reaching wing margin just behind R_3+4_ branch; Rs shorter than both R_3+4_ and R_3_; vein R_3+4_ equal in length to R_3_; crossvein r–r (R_2_) almost halfway along R_1_; d-cell small, slightly widened at apex, twice as long as wide; crossvein m-cu before fork of Mb into M_1+2_ and M_3+4_; A_1_ relatively elongate and nearly straight; A_2_ arched at tip.

*Legs* (Fig. 3A): covered with elongate, not numerous setae.

*Hypopygium* (Fig. 3B–D): gonocoxite elongate, 2.5 × as long as wide, covered with setae, with a bundle of elongate setae at apex; outer gonostylus widely forked into three arms: upper arm slender and widened at the tip, middle arm wider at base and tapers towards the tip, third arm arched at apex; inner gonostylus long, significantly widened at end, terminates in long, thick bristle; straight aedeagus, very wide at base and tapers towards the tip.

Subfamily: Limoniinae Speiser, 1909.

Genus: *Dicranomyia* Stephens, 1829^24^.

Subgenus: *Dicranomyia* Stephens, 1829^24^.

*Type species: Limnobia modesta* Meigen, 1818^15^; by subsequent designation of Coquillett (1910: 533)^17^.

**Fig. 4:**
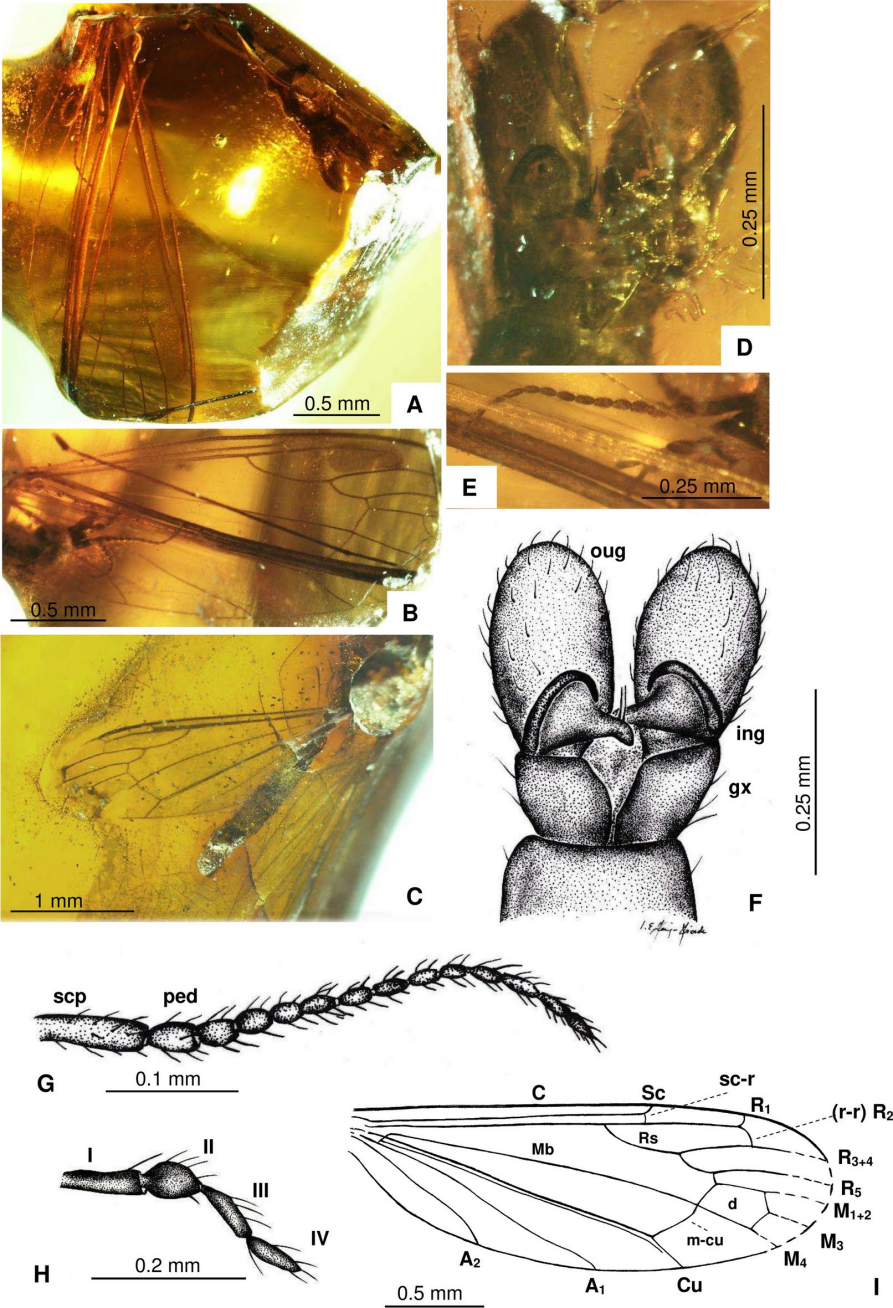
*Dicranomyia* (*Dicranomyia*) *podenasi* Kopeć, Krzemiński & Kania-Kłosok sp. nov., (**A**,**B**,**D**–**I**) holotype MNHN.F.A.97853 (male): (**A**) Habitus, latero-ventral view; (**B**) Wing; (**C**) Additional material MNHN.F.A.97854 (male), habitus; (**D**) Hypopygium, dorsal view; (**E**) Antenna; (**F**) Hypopygium, dorsal view, drawing; (**G**) Antenna, drawing; (**H**) Palpus, drawing; (**I**) Wing, drawing.

*Dicranomyia* (*Dicranomyia*) *podenasi* Kopeć, Krzemiński & Kania-Kłosok sp. nov. (Fig. 4A–I).

LSID urn:lsid:zoobank.org:act:4CF549BB-BDE2-43ED-8D00-C70B01144CC9

#### Diagnosis

Flagellomeres oval and elongate; vein Sc reaching half the length of Rs; Rs as long as R_2+3+4_; m-cu straight, just beyond fork of Mb; pterostigma present; outer gonostylus large, massive, and ballon-like, two slightly elongate spines on rostral prolongation of outer gonostylus.

#### Remarks

Diagnostic features, including simple antennae with elongate, oval flagellomeres that usually narrow toward the apex, a relatively short Sc vein reaching half the length of Rs, two terminal pairs of gonostyles, and a large, ballon-like inner gonostylus with an additional lateral split, place this species in the subgenus *Dicranomyia*.

#### Etymology

The specific name is named in honor of the renowned entomologist, Professor Sigitas Podenas (Vilnius University; Nature Research Centre, Vilnius, Lithuania).

#### Material examined

Holotype,

MNHN.F.A.97853 (male), additional material, MNHN.F.A.97854 (male), housed in the Muséum national d’Histoire naturelle, Paris, France.

#### Remarks

Specimen MNHM,F.A.97853 (holotype) is partially damaged, with incompletely preserved wings apices. Specimen MNHN.F.A.97854 (additional material) is poorly preserved, missing the anterior part of the body, legs, and parts of the wings.

#### Age and occurrence

Eocene, Oise (French) amber, (ca. 55–53 Ma) (Early Eocene, Ypresian, Sparnacian)^18–21^, during the Eocene Thermal Maximum ETM-2 and subsequent hyperthermals^22,23^.

#### Description

Body small (Fig. 4A,C), approximately 2 mm long, wing length 2.4 mm. Head and thorax dark, almost black.

*Head*: antenna (Fig. 4E,G) short, 14 antennomeres; scape elongate and cylindrical, pedicel wide and barrel-like; flagellomeres oval, slightly elongate, taper toward the apex; each flagellomere bears four elongate setae, shorter than antennomere bearing them; palpus (Fig. 4E) moderately elongate, last palpomere approximately equal in length to penultimate one, second palpomere massive and wider than the others (Fig. 4H).

*Wings* (Fig. 4I): vein Sc reaches approximately 0.6 × length of wing; sc-r close to the end of Sc, one length of sc-r before its termination; R_1_ relatively short, reaching wing margin slightly before fork of R_2+3+4_; vein Rs shorter than vein R_3_; crossvein r-r (R_2_) slightly arched, at 0.6 × the length of d-cell; d-cell small, relatively wide and short, expanded in its apical part, 1.5 × as long as wide; crossvein m-cu straight, just beyond fork of Mb into M_1+2_ and M_3+4_; A_1_ relatively elongate and slightly waved; A_2_ short and slightly arched at its tip.

*Legs* (Fig. 4-C, G): covered with elongate, not numerous setae.

*Hypopygium* (Fig. 4D,F): gonocoxite rather short, approximately 1.5 × as long as wide, shorter than outer gonostylus; outer gonostylus massive with sparse, short setae, which are more numerous along its outer edge, two short, moderately robust spines on rostral prolongation of outer gonostylus; inner gonostylus strongly sclerotized and hooked.

## Discussion

Specimens of *Cheilotrichia* found in Oise amber, housed at the Muséum national d’Histoire naturelle in Paris, have been identified as two new species. This discovery extends the known evolutionary history of the genus *Cheilotrichia* by several tens of millions of years. Representatives of the genus *Cheilotrichia* are small flies, typically ranging in size from 2 to 6 mm. These are aquatic insects whose larvae currently inhabit slow-flowing or stagnant waters, as well as very humid substrates. The genus *Cheilotrichia* is divided into two subgenera: *Cheilotrichia* and *Empeda*. The subgenus *Cheilotrichia* comprises 18 extant species, predominantly distributed in the Western Palearctic region. In contrast, the subgenus *Empeda* includes 103 extant species, found across all continents except Antarctica. To date, the oldest representative of this genus was described from Sakhalin amber in eastern Russia, dating to the Middle Eocene^25^. Moreover, this genus has been recorded in Baltic, Ukrainian, and Bitterfeld amber^26–30^, as well as in sedimentary rocks of the Isle of Wight^31,32^. Currently, 20 fossil species of *Cheilotrichia* are known, 17 of which belong to the subgenus *Empeda* and three to the subgenus *Cheilotrichia* (Table 1).

**Table 1:** List of described fossil *Dicranomyia* species.

Species	Age	Locality
*D. antennifera* Théobald, 1937	Oligocene, Chattian	Aix-en-Provence, France
*D. bellissima* Krzemiński, 2001	Eocene, Ypresian	Denmark
*D. exhumata* Cockerell, 1922	Eocene, Priabonian	Isle of Wight, UK
*D. faecaria* Scudder, 1894	Eocene, Priabonian	Florissant, USA
*D. fontainei* Scudder, 1894	Eocene, Priabonian	Florissant, USA
*D. fragilis* Scudder, 1894	Eocene, Priabonian	Florissant, USA
*D. freiwaldi* Krzemiński, 2001	Eocene, Ypresian	Denmark
*D. inferna* Scudder, 1894	Eocene, Priabonian	Florissant, USA
*D. loewi* Scudder, 1894	Eocene, Priabonian	Florissant, USA
*D. longipes* Scudder, 1894	Eocene, Priabonian	Florissant, USA
*D. primitiva* Scudder, 1877	Eocene, Ypresian-Lutetian	Utah, USA
*D. primoriana* Krzemiński, 2000	Eocene, Priabonian	Primorye, Russia
*D. rhodolitha* Cockerell, 1908	Eocene, Ypresian-Lutetian	Wyoming, USA
*D. rohweri* Brown, 1985	Eocene, Priabonian	Florissant, USA
*D. rostrata* Scudder, 1877	Eocene, Ypresian-Lutetian	Utah, USA
*D. saxetana* Scudder, 1894	Eocene, Priabonian	Florissant, USA
*D. sergio* Krzemiński & Gentilini, 1992	Miocene, Messinian	Monte Castellaro, Italy
*D. stagnorum* Scudder, 1894	Eocene, Priabonian	Florissant, USA
*D. speciosa* Krzemiński, 2001	Eocene, Ypresian	Denmark
*D. stigmosa* Scudder, 1877	Eocene, Ypresian-Lutetian	Utah, USA
*D. thybotica* Henriksen, 1922	Eocene, Ypresian	Denmark
*D. undulata* Cockerell & Haines, 1921	Eocene, Priabonian	Isle of Wight, UK
*D.* (*Caenolimonia*) *alexbrowni* Podenas & Poinar, 2012	Miocene	Chiapas, Mexico
*D.* (*Dicranomyia*) *alexandri* Kania, 2013	Eocene, Lutetian	Baltic Region
*D.* (*D.*) *aliena* (Cockerell, 1922)	Eocene, Priabonian	Isle of Wight, UK
*D.* (*D.*) *baltica* Kania, 2014	Eocene, Lutetian	Baltic Region
*D.* (*D.*) *chiapa* Podenas & Poinar, 2012	Miocene	Chiapas, Mexico
*D. (D.) colombiana* Krzemiński & Kania, 2018	Pleistocene, Calabrian	Colombia
*D.* (*D.*) *ewa* Kania, 2014	Eocene, Lutetian	Baltic Region
*D.* (*D.*) *fera* Podenas & Poinar, 1999	Miocene, Burdigalian	Dominican Republic
*D.* (*D.*) *gorskii* Kania, Krzemiński & Penar, 2013	Eocene, Lutetian	Baltic Region
*D.* (*D.*) *graciosa* Meunier, 1916	Eocene, Lutetian	Baltic Region
*D.* (*D.*) *grandis* Meunier, 1899	Eocene, Lutetian	Baltic Region
*D.* (*D.*) *indica* Kania, Krzemiński, Stebner & Singh, 2018	Eocene, Ypresian	India
*D.* (*D.*) *kalandyki* Krzemiński, 2000b	Eocene, Lutetian	Baltic Region
*D.* (*D.*) *lema* Podenas & Poinar, 1999	Miocene, Burdigalian	Dominican Republic
*D.* (*D.*) *lobata* Meunier, 1906	Eocene, Lutetian	Baltic Region
*D.* (*D.*) *meunieri* Alexander, 1931	Eocene, Lutetian	Baltic Region
*D.* (*D.*) *mexa* Podenas & Poinar, 2012	Miocene	Chiapas, Mexico
*D.* (*D.*) *perpendicularis* Savchenko, 1967	Eocene, Lutetian	Baltic Region
*D. (D.) podenasi* Kopeć, Krzemiński & Kania-Kłosok sp. nov	Eocene, Ypresian	Oice, France
*D.* (*D.*) *sinuata* Meunier, 1916	Eocene, Lutetian	Baltic Region
*D.* (*D.*) *succinica* Kania, 2014	Eocene, Lutetian	Baltic Region
*D.* (*D.*) *vella* Podenas & Poinar, 2012	Miocene	Chiapas, Mexico
*D.* (*Melanolimonia*) *krzeminskii* Kania, 2014	Eocene, Lutetian	Baltic Region
*D.* (*M.*) *kukulai* Krzemiński, Kania & Wojtoń, 2018	Eocene, Lutetian	Baltic Region
*D.* (*Sivalimnobia*) *herczeki* Krzemiński & Kania, 2015	Eocene, Lutetian	Baltic Region

The second identified genus, *Dicranomyia*, is currently one of the most diverse genera within Limoniidae, comprising as many as 1136 extant species. It is also well-represented among fossil flies, with 45 species described to date. Extinct representatives of *Dicranomyia* are known from various geological periods—primarily the Eocene, but also the Oligocene and Miocene—and from multiple continents. The oldest representatives of the genus *Dicranomyia* were described from the early Eocene Mo Clay (Für Formation) in Denmark^33,34^ (Table 2). To date, 16 species from Eocene resins have been described: one from Early Eocene Indian amber (ca. 54 Ma) and 15 from Eocene Baltic amber^28,30,35–41^. An additional 17 Eocene species are known from rock impressions: one from tuff layers at the Bolshaya Svetlovodnaya River, Russia^42^; four from the Green River Formation, USA^43,44^; three from the Upper Eocene Bembridge Marls of England^31,45,46^; and nine from Florissant, USA^46,47^ (Table 2). Three subgenera of *Dicranomyia* have been recorded in Baltic amber, with *Dicranomyia* being the most common and diverse in the extant fauna. Representatives of the other subgenera in Baltic amber are rare and are represented by only a few species: *Dicranomyia* (*Melanolimonia*) *krzeminskii* Kania, 2014^39^, *D*. (*M*.) *kukulai* Krzemiński, Kania & Wojtoń, 2018^48^, and *Dicranomyia* (*Sivalimnobia*) *herczeki* Krzemiński & Kania, 2015^41^. Gelhaus & Johnson^29^ described the species *Limonia* (*s. lato*) *dillone* from New Jersey amber (Upper Cretaceous) based on a female specimen. This species likely belongs to *Dicranomyia* and may represent its oldest known member.

**Table 2:** List of described fossil *Cheilotrichia* species.

Species	Age	Locality
*Ch.* (*Cheilotrichia*)* antiqua* Podenas, 1999	Eocene, Lutetian	Baltic Region
*Ch.* (*Cheilotrichia*) *duplicata* Krzemiński, 2019	Eocene, Priabonian	Isle of Wight, UK
*Ch.* (*Cheilotrichia*) *gilija* Podenas, 1999	Eocene, Lutetian	Baltic Region
*Ch.* (*Empeda*) *axillaris* Alexander, 1931	Eocene, Lutetian	Baltic Region
*Ch.* (*Empeda*) *bella* Podenas, 1999	Eocene, Lutetian	Baltic Region
*Ch.* (*Empeda*) *budrysi* Podenas, 1999	Eocene, Lutetian	Baltic Region
*Ch.* (*Empeda*) *cretacea* Gelhaus & Johnson, 1996	Upper Cretaceous, Turonian	New Jersey, USA
*Ch.* (*Empeda*) *diacantha* Alexander, 1931	Eocene, Lutetian	Baltic Region
*Ch.* (*Empeda*) *duplicata* Alexander, 1931	Eocene, Lutetian	Baltic Region
*Ch. (Empeda) ferruginea* (Cockerell, 1921)	Eocene, Priabonian	Isle of Wight, UK
*Ch*. (*Empeda*) *gallica* Kopeć, Krzemiński & Kania-Kłosok sp. nov	Eocene, Ypresian	Oise, France
*Ch.* (*Empeda*) *hyalina* (Cockerell, 1921)	Eocene, Priabonian	Isle of Wight, UK
*Ch.* (*Empeda*) *minuta* (Meunier, 1899)	Eocene, Lutetian	Baltic Region
C*h.* (*Empeda*) *oisensis* Kopeć, Krzemiński & Kania-Kłosok sp. nov	Eocene, Ypresian	Oice, France
*Ch.* (*Empeda*)* palaeocenica* Krzemiński & Krzemińska, 1994	Eocene, Lutetian	Sakhalin, Russia
*Ch.* (*Empeda*) *pawlowskii* Kopeć, 2019	Eocene, Lutetian	Baltic Region
*Ch.* (*Empeda*) *platyphylla* Alexander, 1931	Eocene, Lutetian	Baltic Region
*Ch.* (*Empeda*) *rectistyla* Alexander, 1931	Eocene, Lutetian	Baltic Region
*Ch.* (*Empeda*) *schummeli* Meunier 1917	Eocene, Lutetian	Baltic Region
*Ch.* (*Empeda*) *subabortiva* Alexander, 1931	Eocene, Lutetian	Baltic Region
*Ch.* (*Empeda*) *szwedoi* Krzemiński, 2019	Eocene, Priabonian	Isle of Wight, UK
*Ch.* (*Empeda*)* weitschati* Kopeć & Kania, 2013	Eocene, Lutetian	Bitterfeld (Saxonian)

**Fig. 5:**
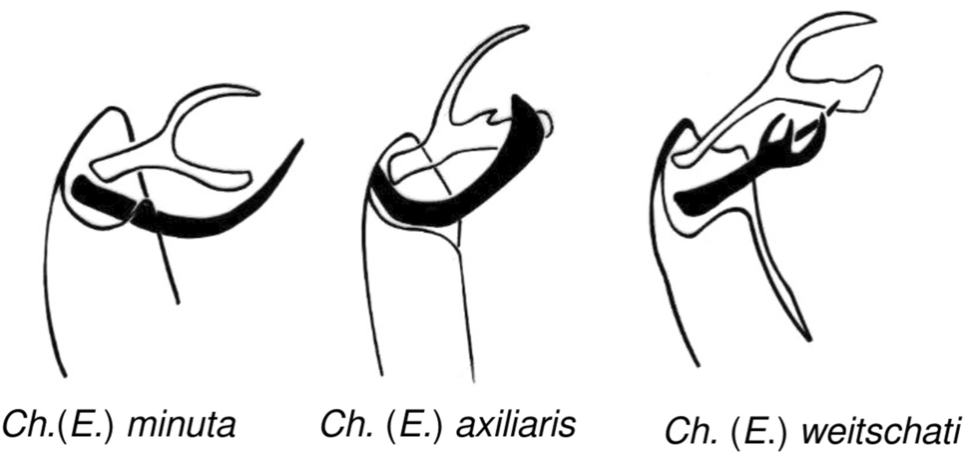
Different types of hypopygium of the subgenus *Empeda*.

**Fig. 6:**
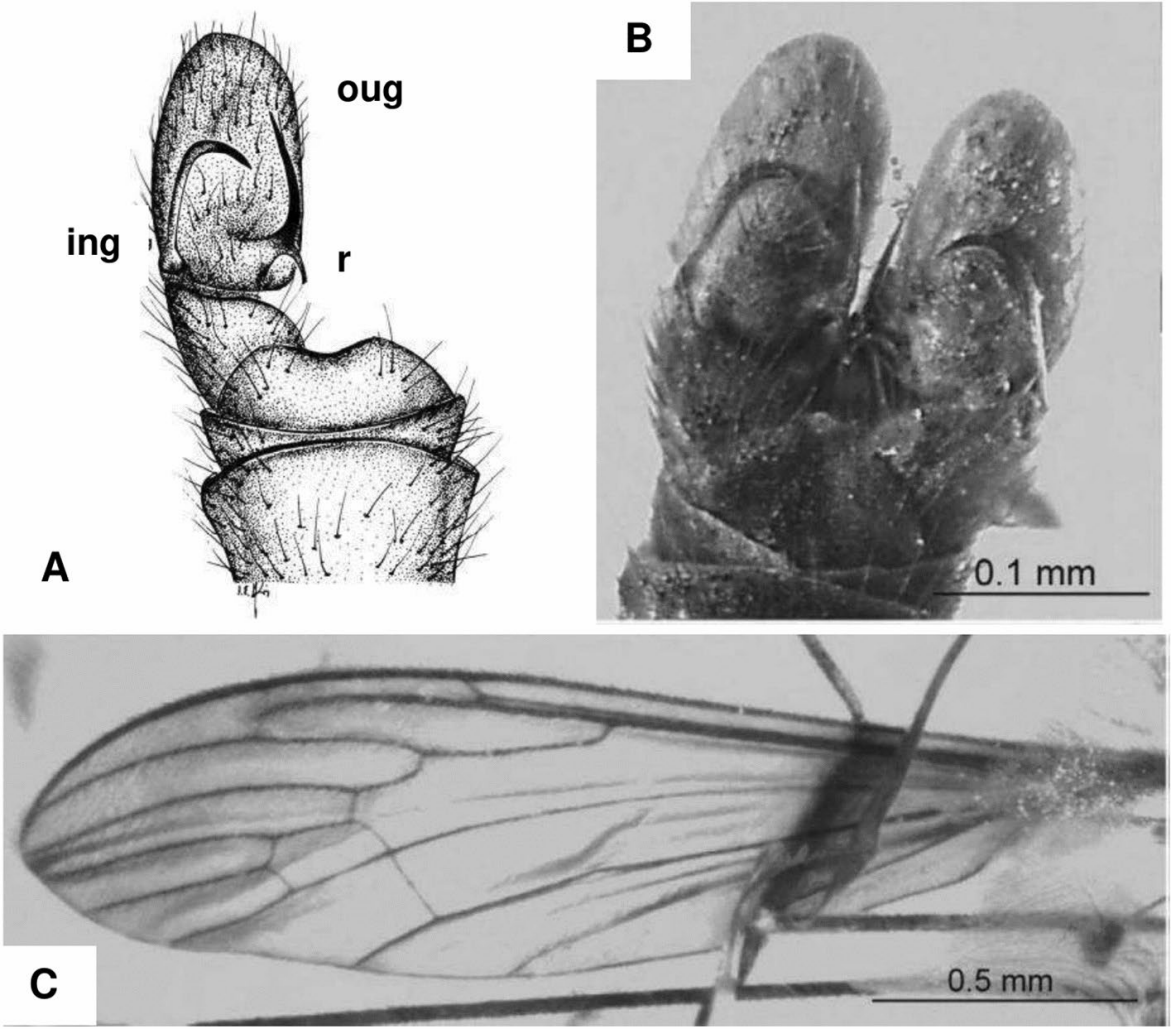
*Dicranomyia gorskii* Kania et al., 2013, (**A**–**C**) holotype No. MP/3313 (6550): (**A**) hypopygium (part), dorsal view, drawing; (**B**) hypopygium, dorsal view, photograph; (**C**) wing (after Kania et al., 2013, changed). *oug* outer gonostylus, *ing* inner gonostylus, *r* rostrum with spine.

The newly described species of *Cheilotrichia* from Oise amber were classified within the subgenus *Empeda*, primarily based on the structure of the male terminalia (Fig. 5). The first of these new species, *Ch.* (*E.*) *oisensis* sp. nov., exhibits a hypopygium structure somewhat similar to that of *Ch*. (*E*.) *minuta* (Fig. 5). However, notable differences are evident in *Ch.* (*E.*) *oisensis* sp. nov.; its outer gonostylus is widely forked. The upper arm of the fork is slender with a sharply pointed tip, whereas the lower arm is significantly widened at the tip and slightly convex in the middle. In contrast, the outer gonostylus of *Ch.* (*E*.) *minuta* resembles a slingshot, with only slight differences between arms. The lower arm is merely slightly widened at the end (Fig. 6). The inner gonostylus also shows striking differences between the two species. In *Ch.* (*E*.) *oisensis* sp. nov., it is strongly widened at the tip and terminates in long, thick bristles. In *Ch.* (*E*.) *minuta*, the inner gonostylus tapers sharply toward the apex and lacks bristles at the tip. Further differences are observed in the wing venation. In *Ch.* (*E*.) *oisensis* sp. nov., the Rs vein is slightly longer than the R_3+4_ vein and exceeds the length of the R_3_ vein by approximately 0.25 × its length. In *Ch.* (*E*.) *minuta*, the Rs vein is almost 50% longer than the R_3+4_ vein and almost equal in length to the R_3_ vein.

In contrast, the second newly described species, *Ch.* (*E.*) *gallica* sp. nov., is most similar to *Ch.* (*E.*) *axiliaris*, but it differs clearly in the structure of both the outer and inner gonostyles (Fig. 5). In *Ch.* (*E.*) *gallica* sp. nov., the outer gonostylus splits into three long processes, with the last one curled toward the second process. In *Ch.* (*E*.) *axiliaris*, however, the middle and last appendages are only slightly outlined. The inner gonostylus in the new species is strongly widened at the tip and ends with long, thick bristles. In contrast, the inner gonostylus of *Ch.* (*E*.) *axiliaris* also tapers to a sharp tip, but it is not as pronounced as in *Ch.* (*E*.) *gallica* sp. nov. and lacks bristles at the tip, featuring instead an additional appendage. Further differences are observed in the wing venation. The most noticeable difference is in the length of the Rs vein. In *Ch.* (*E*.) *gallica* sp. nov., the Rs vein is shorter than both the R_3+4_ and R_3_ veins, whereas in *Ch.* (*E*.) *axiliaris*, the Rs vein is longer than both.

Of the 36 *Dicranomyia* species known from the Eocene to date, the newly described species *D.* (*D.*) *podenasi* sp. nov. is most similar in wing venation to *D.* (*D.*) *sinuata*. The new species exhibits characteristics of the subgenus *Dicranomyia* and has been classified accordingly. Three of the remaining 36 Eocene species belong to other subgenera: *Melanolimonia* and *Sivalimnobia*. Despite similarities in wing venation, *D.* (*D.*) *podenasi* exhibits several differences when compared to *D.* (*D.*) *sinuata*. The crossvein m-cu in the new species is not as strongly sinuous, but rather straight, and it does not originate directly from the Mb fork but just behind it. Additionally, *D.* (*D.*) *podenasi* lacks a pterostigma, and the crossvein r-r (R_2_) is more curved than in *D.* (*D.*) *sinuata*. There are also notable differences in the structure of the hypopygium. In *D.* (*D.*) *podenasi*, the outer gonostylus is very large, while the inner gonostylus is strongly sclerotized and hook-like and bent towards the interior of the hypopygium. The relative proportions of the outer gonostyles and inner gonostyles in this species distinguish it from *D.* (*D.*) *sinuata*. The prominent outer gonostylus in *D.* (*D.*) *podenasi* is a distinctive feature that sets it apart from *D.* (*D.*) *sinuata*. A similar gonocoxite structure is found in *D.* (*D.*) *gorskii* (Fig. 6), but in this species, there is a single very elongate spine on the rostral prolongation of the outer gonostylus. In contrast, *D.* (*D.*) *podenasi* is characterized by two less elongate spines on the rostral prolongation of the outer gonostylus. A very large outer gonostylus is also present in *D.* (*D*.) *ewa*, but this species features a single very elongate, strongly sclerotized spine on the rostral prolongation of the outer gonostylus, which clearly distinguishes it from the *D.* (*D.*) *podenasi* sp. nov. Moreover, the vein Sc in *D.* (*D.*) *ewa* is much longer than in *D.* (*D.*) *podenasi* sp. nov., almost reaching fork of Rs. *D.* (*D.*) *graciosa* also has two less elongate spines on the rostral prolongation of the outer gonostylus, but the very large outer gonostylus of this species differs in shape from that of *D.* (*D.*) *podenasi* sp. nov. In *D.* (*D.*) *podenasi* sp. nov., the tip of the outer gonostylus is very wide, while in *D.* (*D.*) *graciosa*, it is somewhat conical. Additionally, in *D.* (*D.*) *podenasi* sp. nov., the Rs vein is very short, about the same length as R_2+3+4_, whereas in *D.* (*D.*) *graciosa*, it is nearly twice as long.

Amber from Oise, specifically from the Quesnoy locality in the Oise River area of the Paris basin (Oise department, France), is rich in both flora and fauna, providing a significant number of fossils trapped in its resin. Oise amber from northern France is the oldest Eocene resin, dating back approximately 55–53 Ma (Early Eocene, Ypresian, Sparnacian)^18–21,51^. This period coincided with the Eocene Thermal Maximum (ETM-2) and subsequent hyperthermals^22,23^. Organic inclusions in Oise amber exhibit excellent preservation, allowing the description of over 80 species from more than 60 insect families, as well as more than 300 morphospecies of arthropods^18,20,51^. Based on direct observations and work with both ambers, Oise amber is softer and more brittle than Baltic amber. Although Baltic and Oise ambers share a similar geographical origin, they were produced at different times and by different plant species: Baltic amber originated from coniferous trees, while Oise amber was produced by *Aulacoxylon sparnacense* Combes, 1907^52^ from the family Fabaceae (Caesalpinioideae; Detariae)^21,52^. The precise age of Baltic amber remains a topic of debate, from early Eocene (dated 47–41 Ma, Lutetian)^49^, to the late Eocene (Priabonian)^50^. These discrepancies in dating are due to the fact that the Baltic amber deposit was redeposited. The forests that produced Oise amber were a mosaic of gallery-forest mixed with drier plant communities, formed by a fluvial system with multiple channels and ponds thriving in a deltaic, paratropical region^20,51,54^. Furthermore, the results of the study on the family Ceratopogonidae from Oise amber by Szadziewski et al.^55^ also reveal the marine influence, as good indicators of seashore or estuarine environments were found in this resin.

The Limoniidae in amber from Oise (France) are represented by only three species, all of which differ from the taxa known from Baltic amber. Unlike Oise amber, the Limoniidae fauna in Baltic amber is very well studied. Based on this, we can assume that the fauna of these flies is likely distinct from that of Baltic amber. Despite the geographical proximity of these deposits, no Limoniidae species have been found in Baltic amber. This suggests significant environmental and evolutionary differences, likely resulting from temporal separation. It is also plausible to hypothesize that the fauna of the Eocene forests in the Oise region differed from that of the forests of Fennoscandia. In this context, comparing the faunal composition of both Eocene ambers is crucial, as the discovery of even a single shared species in both ambers would suggest a similar age (given that insect species are estimated to persist for up to 5 million years^56^). Further research on Oise amber will undoubtedly help verify this hypothesis.

## Material and methods

This research is based on material from the collection of the Muséum national d’Histoire naturelle, Paris, France, consisting of six specimens originating from amber deposits in Oise, northern France. The examined material included four specimens of flies from the genus *Cheilotrichia*, identified into two new species, and two specimens from the genus *Dicranomyia*, dentified as one species. Representatives of the new species were studied using a Nikon SMZ 1500 stereomicroscope. Photographs were taken with a Nikon DS-Fi1 camera mounted on the microscope. Measurements were recorded using NIS-Elements D 3.0 software, focusing exclusively on undamaged body parts. Measurements such as d-cell length were taken from its posterior edge to the point where vein m-m connects with vein M_3_.

The wing venation nomenclature follows Krzemiński & Krzemińska^57^, while terminology for the hypopygium structures is based on McAlpine et al.^58^. Illustrations were prepared using both direct observations of specimens and photographs.

**Fig. 7:**
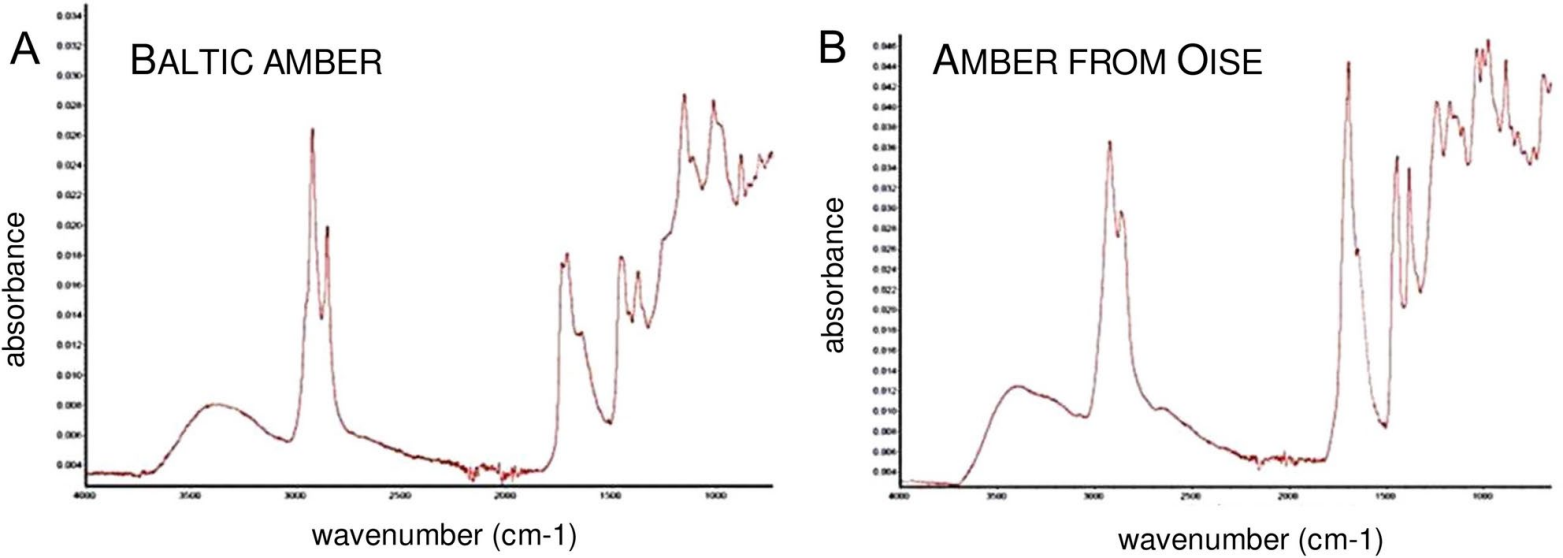
Infrared spectra of Baltic (**A**) and Oise amber (**B**).

The FT-IR spectrum analysis (Fig. 7A,B) was performed at the Institute of Systematics and Evolution of Animals, Polish Academy of Sciences in Kraków, Poland. A Nicolet iS10 spectrometer equipped with an ATR (Attenuated Total Reflectance; diamond crystal) accessory was used to obtain spectra at resolution of 4 cm^−1^. The FT-IR method, widely employed to classify fossilized resins, is sufficient to identify amber in most cases^59,60^. Map these tests confirmed the type of amber. Oise amber was previously chemically characterized by Jossang et al.^61^. Paper was registered in ZooBank LSID urn:lsid:zoobank.org:pub:5150E03D-AEBD-47CD-8784-93607FDF245E

The map of the Eocene outcrops (Fig. 1) was created using the website https://paleobiodb.org/navigator/, which allows users to “copy, modify, distribute, and perform the work, even for commercial purposes, all without asking permission” under the CC0 1.0 Universal Public Domain Dedication license.

## Data Availability

All data generated or analysed during this study are loaded in this published article.

## References

[1] Oosterbroek, P. *CCW: Catalogue of the Craneflies of the World (Diptera, Tipuloidea: Pediciidae, Limoniidae, Cylindrotomidae, Tipulidae)* https://ccw.naturalis.nl/index.php (2024).

[2] Krzemiński, W., Kania-Kłosok, I., Krzemińska, E., Ševčík, J. & Soszyńska-Maj, A. Fossils shed a new light on the diversity and disparity of the Family Limoniidae (Diptera, Nematocera). *Insects* **12**(3), 206. 10.3390/insects12030206 (2021). 33804356 10.3390/insects12030206 PMC8000698

[3] Rohdendorf, B. B. Historical development of dipterous insects. *Trudy Paleontol. Inst. AN SSSR.* **100**, 1–311 (1964) (**In Russian, English translation in Rohdendorf, 1974**).

[4] Krzemiński, W. & Zessin, W. The Lower Jurassic Limoniidae from Grimmen (GDR). *Dtsch. Entomol. Z.* **37**, 39–43 (1990).

[5] Oberprieler, S. K., Krzemiński, W., Hinde, J. & Yeates, D. K. First crane fly from the Upper Jurassic of Australia (Diptera: Limoniidae). *Zootaxa* **4021**(1), 178–186 (2015). 26624125 10.11646/zootaxa.4021.1.8

[6] Kopeć, K., Krzemiński, W., Skowron, K. & Coram, R. The genera *Architipula* Handlirsch, 1906 and *Grimmenia* Krzemiński and Zessin, 1990 (Diptera: Limoniidae) from the Lower Jurassic of England. *Palaeontol. Electron.* **20.1.15A**, 1–7. 10.26879/637 (2017).

[7] Kopeć, K., Perkovsky, E. & Skibińska, K. A new species of a genus *Cheilotrichia* (Diptera: Limoniidae) from Baltic and Ukrainian amber. *Ann. Zool.* **69**, 423–426 (2019).

[8] Kopeć, K., Ansorge, J., Soszyńska-Maj, A. & Krzemiński, W. Revision of the genus *Mesotipula* Handlirsch, 1920 (Diptera, Limoniidae, Architipulinae) from the Lower Jurassic of Northeast Germany. *Hist. Biol.* **32**(4), 5000507. 10.1080/08912963.2018.1503257 (2018).

[9] Kopeć, K., Soszyńska-Maj, A., Lukashevich, E. & Krzemiński, W. Revision of the Mesozoic genus *Mesotipula* Handlirsch (Limoniidae, Diptera) from Asia extending its evolutionary history up to the Cretaceous. *Cretac. Res.* **114**(1), 104504 (2020).

[10] Kopeć, K., Soszyńska-Maj, A., Kania-Kłosok, I., Coram, R. A. & Krzemiński, W. Morphology of the oldest fossil subfamily of Limoniidae (Diptera, Architipulinae) in the light of exceptionally preserved Mesozoic material. *Sci. Rep.* **11**, 24137. 10.1038/s41598-021-03350-4 (2021). 34921169 10.1038/s41598-021-03350-4 PMC8683464

[11] Krzemiński, W. et al. The evolutionary history and biogeographical distribution of the Mesozoic relic genus *Chilelimnophila* (Diptera, Limoniidae). *Zool. J. Linn. Soc.* **202**, 119. 10.1093/zoolinnean/zlae119 (2024).

[12] Handlirsch, A. *Die fossilen Insecten und die Phylogenie der rezent Formen* (Engelmann, 1906–1908).

[13] Alexander, C. P. New or little-known Tipulidae from Chile and Peru (Diptera: Tipulidae). *Rev. Chil. Entomol.* **6**, 21–36 (1968).

[14] Rossi, F. W. *Systematisches Verzeichniss der zweiflugelichten Insecten (Diptera) des Erzherzogthumes Osterreich mit Angabe des Standortes, der Flugzeit und einigen andern physiologischen Bemerkungen* 1–86 (W. Braumuller, 1848).

[15] Meigen, J. W. *Systematische Beschreibung der bekannten europaischen zweiflugeligen Insecten.* T.1. (FW. Forstmann, 1818).

[16] Osten Sacken, C. R. New genera and species of North American Tipulidae with short palpi, with an attempt at a new classification of the tribe. *Proc. Acad. Nat. Sci. Phila.* **11**, 197–256 (1860).

[17] Coquillett, D. W. The type species of the North American genera of Diptera. *Proc. U. S. Natl. Mus.* **37**, 499–647 (1910).

[18] Feugueur, L. L’Yprésien du bassin de Paris. Essai de monographie stratigraphique. *Mém. serv. explic. carte géol.* Fr. 1–568 (Imprimerie National, 1963).

[19] Nel, A. et al. Un gisement sparnacien exceptionnel à plantes, arthropodes et vertébrés (Éocène basal, MP7): Le Quesnoy (Oise, France). *C. R. Acad. Sci.*, *Ser. IIa, Sci. Terre Planètes* **329**, 65–72 (1999).

[20] Nel, A., De Ploëg, G., Milliet, J., Menier, J.-J. & Waller, A. The French ambers: a general conspectus and the Lowermost Eocene amber deposit of Le Quesnoy in the Paris Basin. *Geol. Acta* **2**, 3–8 (2004).

[21] De Franceschi, D. & De Ploëg, G. Origine de l’ambre des faciès sparnaciens (Éocène inférieur) du Bassin de Paris: le bois de l’arbre producteur. *Geodiversitas* **25**, 633–647 (2003).

[22] Lourens, L. J. et al. Astronomical pacing of Late Palaeocene to Early Eocene global warming events. *Nature* **435**, 1083–1087 (2005). 15944716 10.1038/nature03814

[23] Stap, L. et al. High-resolution deep-sea carbon and oxygen isotope records of Eocene Thermal Maximum 2 and H2. *Geology* **38**, 607–610 (2010).

[24] Stephens, J. F. *The Nomenclature of British Insects; Being a Compendious List of Such Species as are Contained in the Systematic Catalogue of British Insects, and Forming a Guide to their Classification* (Baldwin & Cradock, 1829).

[25] Krzemiński, W. & Krzemińska, E. A new species of *Cheilotrichia* (*Empeda*) from the Sakhalin amber (Diptera, Limoniidae). *Acta Zool. Cracov.* **37**, 91–93 (1994).

[26] Kopeć, K. & Kania, I. A new species of *Cheilotrichia* Rossi, 1848 (Diptera: Limoniidae) from Bitterfeld amber. *Ann. Zool.* **63**, 537–540 (2013).

[27] Podenas, S. New *Cheilotrichia* crane flies (Diptera, Limoniidae) from Baltic amber. *Mitt. Geol.-Palaontol. Inst. Univ. Hamb.* **83**, 239–248 (1999).

[28] Alexander, C. P. Crane flies of the Baltic Amber (Diptera). *Bernstein-Forschungen.* **2**, 1–135 (1931).

[29] Gelhaus, J. & Johnson, R. First record of crane flies (Tipulidae: Limoniinae) in Upper Cretaceous amber from New Jersey, U.S.A. *Trans. Am. Entomol. Soc.* **122**, 55–65 (1996).

[30] Meunier, F. Révision des diptères fossiles types de Loew conservés au Musée Provincial de Koenigsberg. *Misc. Entomol.* **7**, 169–182 (1899).

[31] Cockerell, T. D. A. New name for a fossil tipulid fly. *Entomologist* **55**, 17 (1922).

[32] Krzemiński, W., Blagoderov, V. & Azar, D. True flies (Insecta: Diptera) from the late Eocene insect limestone (Bembridge Marls) of the Isle of Wight, England, UK. *Earth Environ. Sci. Trans. R. Soc. Edinb.* **110**, 495–554 (2019).

[33] Henriksen, K. L. Eocene insects from Denmark. *Danmarks Geologiske Undersøgelse II. Række* **37**, 1–36 (1922).

[34] Krzemiński, W. New fossil Tipuloidea (Diptera) from the Für Formation of Denmark in the collection of the Natural History Museum in London. *Pol. J. Entomol.* **70**, 333–339 (2001).

[35] Meunier, F. Monographie des Tipulidae et Dixidae de l’ambre de la Baltique. *Ann. Sci. Nat. Zool.* **4**, 349–401 (1906).

[36] Meunier, F. Beitrag zur Monographie des Tipuliden des Bernsteins. *Z. Dtsch. Geol. Ges. (A)* **68**, 477–493 (1916).

[37] Savchenko, E. N. New fossil limoniid fly (Diptera, Limoniidae) from the Baltic amber. *Dopov. Akad. Nauk URSR*(B). **5**, 469–473 (1967).

[38] Krzemiński, W. A new species and other representatives of the genus *Dicranomyia* (Diptera: Limoniidae) in the collection of the Museum of Amber Inclusions, University of Gdańsk. *Pol. J. Entomol.* **69**(3), 347–353 (2000).

[39] Kania, I. Subfamily Limoniinae Speiser, 1909 (Diptera, Limoniidae) from Baltic amber (Eocene). The genus Dicranomyia Stephens, 1829. *Zool. J. Linn. Soc.* **170**, 748–778 (2014).

[40] Kania, I., Krzemiński, W. & Penar, A. A new species of *Dicranomyia* (*Dicranomyia*) Stephens, 1829 from Baltic amber (Diptera: Limoniidae). *Ann. Zool.* **63**, 143–148 (2013).

[41] Krzemiński, W., Kania, I. & Durak, R. A new species of *Dicranomyia* Stephens, 1829 (Diptera: Limoniidae) from Baltic amber (Eocene). *Neues Jahrb. Geol. Paläontol. Abh.* **277**(2), 167–174 (2015).

[42] Krzemiński, W. The Oligocene Tipulomorpha (Diptera) from Bolshaya Svetlovodnaya (Eastern Asia, Russia). *Pol. J. Entomol.* **69**(2), 239–245 (2000).

[43] Scudder, S. H. The first discovered traces of fossil insects in the American tertiaries. *Bull. U. S. Geol. Geogr. Surv. Territories.* **3**, 741–42 (1877).

[44] Cockerell, T. D. A. Descriptions of Tertiary insects. Part II. *Am. J. Sci.* **25**, 227–232 (1908).

[45] Cockerell, T. D. A. & Haines, F. H. Fossil Tipulidae from the Oligocene of the Isle of Wight, continued. *Entomologist* **54**, 109–112 (1921).

[46] Scudder, S. H. Tertiary Tipulidae, with special reference to those of Florissant. *Colorado. Proc. Am. Philos. Soc.* **32**, 163–245 (1894).

[47] Brown, F. M. Four undescribed Oligocene craneflies from Florissant, Colorado, (Diptera: Tipulidae). *Insecta Mundi* **1**, 98–100 (1985).

[48] Krzemiński, W., Kania, I. & Wojtoń, M. A new Eocene *Dicranomyia* Stephens, 1829 (Diptera: Limoniidae) from Baltic amber. *Earth Environ. Sci. Trans. R. Soc. Edinb.* **107**, 271–277 (2018).

[49] Ritzkowski, S. K-Ar-Altersbestimmungen der bernsteinführenden Sedimente des Samlands (Paläogen, Bezirk Kaliningrad). *Metalla* **66**, 19–23 (1997).

[50] Grimaldi, D. & Ross, A. J. Extraordinary Lagerstatten in amber, with particular reference to the Cretaceous of Burma. In *Terrestrial Conservation Lagerstatten: Windows into the Evolution of Life on Land* (eds Fraser, N. C., Sues, H. D.) 303 (*Dunedin Academic Press*, 2017).

[51] Nel, A. & Brasero, N. Oise amber in Biodiversity of fossils in amber from the major world deposits (ed. Penney, D.) 137–148 (Siri Scientific Press, 2010).

[52] Combes, P. Contribution a l’étude de la Flore Éocène. Sur un bois fossile nouveau appartenant a l’étage Sparnacien. *Bull. Soc. Geol. Fr.* **4**, 28–29 (1907).

[53] Wolfe, A. P. et al. A new proposal concerning the botanical origin of Baltic amber. *Proc. R. Soc. B* **276**, 3403–3412 (2009). 19570786 10.1098/rspb.2009.0806 PMC2817186

[54] Drohojowska, J. & Szwedo, J. The first Aleyrodidae from the Lowermost Eocene Oise amber (Hemiptera: Sternorrhyncha). *Zootaxa* **3636**, 319–347 (2013). 26042295 10.11646/zootaxa.3636.2.5

[55] Szadziewski, R., Santer, M., Nel, A., Krzemińska, E., Soszyńska, A. et al. Biting midges (Diptera: Ceratopogonidae) from Lower Eocene amber of Oise, France have no faunal connection with other Eocene faunas. *Acta Palaeontol. Pol.* (in press).

[56] Simpson, G. G. The species concept. *Evolution* **5**, 285–298 (1951).

[57] Krzemiński, W. & Krzemińska, E. Triassic Diptera: descriptions, revisions and phylogenetic relations. *Acta Zool. Cracov.* **46**(suppl. Fossil Insects), 153–184 (2003).

[58] McAlpine, J. F. B. et al. Manual of Nearctic Diptera. *Agric. Can. Res. Branch Monogr.* **1**, 27–674 (1981).

[59] Kosmowska-Ceranowicz, B. Atlas widm w podczerwieni żywic kopalnych, subfosylnych i niektórych imitacji bursztynu. In *Widma IR żywic kopalnych/Charakterystyka ich holotypów* (ed. Kosmowska-Ceranowicz, B.) 213 (Wydawnictwo Muzeum Ziemi PAN, 2015).

[60] Kosmowska-Ceranowicz, B., Wagner-Wysiecka, E. & Całka, S. Diagnostyczne pasma IRS po modyfikacji bursztynu. *Pr. Muz. Ziemi* **50**, 57–65 (2012).

[61] Jossang, J., Bel-Kassaoui, H., Jossang, A., Seuleiman, M. & Nel, A. Quesnoine, a Novel Pentacyclic *ent*-Diterpene from 55-Million Years Old Oise Amber. *J. Org. Chem.* **73**, 412–417 (2008). 18154348 10.1021/jo701544k

